# Asymptomatic microscopic colitis induced by secukinumab

**DOI:** 10.1016/j.jdcr.2024.03.017

**Published:** 2024-04-09

**Authors:** Matteo Megna, Mario De Lucia, Luigi Fornaro, Nello Tommasino, Fabiana Castiglione, Sara Cacciapuoti, Anna Testa

**Affiliations:** a Dermatology, Department of Clinical Medicine and Surgery, University of Naples Federico II, Naples, Italy; b Gastroenterology, Department of Clinical Medicine and Surgery, University of Naples Federico II, Naples, Italy

**Keywords:** anti IL-17, microscopic colitis, psoriasis, secukinumab

## Introduction

Interleukin-17 (IL-17) inhibitors are effective in treating chronic inflammatory diseases such as psoriasis and psoriatic arthritis (PsA).^1^ Secukinumab, an anti-IL-17 monoclonal antibody, is 1 such approved treatment.^1^ Thus, rare cases of flare-ups or new onset inflammatory bowel disease (IBD), including ulcerative colitis and Crohn's disease, have been occasionally reported under secukinumab use.^1^ Microscopic colitis (MC) is a long-term inflammatory bowel condition characterized by chronic watery diarrhea and normal or mild findings in colonoscopy with specific pathological features.^2^ It can consist of collagenous colitis or lymphocytic colitis.^2^ Risk factors include being female over 50 years old, smoking and certain medications, including non-steroidal anti-inflammatory drugs, proton-pump inhibitors, and selective serotonin reuptake inhibitors.^3^ This report describes the case of a 58-year-old male patient affected by PsA and psoriasis developing asymptomatic MC under secukinumab.

## Case presentation

A 58-year-old man with psoriasis and PsA unresponsive to methotrexate presented to our outpatient clinic for the abrupt development of erythematous plaques with whitish scale on his trunk and limbs. Dermatological examination showed an extension of these lesions affecting a body surface area of 30%. He also had peripheral PsA mainly involving his hands and feet. His medical history was unremarkable, except for hypertension, and he did not smoke. Routine laboratory investigations including thyroid function, celiac disease, and autoantibodies were normal. The patient had no personal or family history of IBD. Secukinumab treatment at recommended dose for psoriasis achieved complete skin clearance within 8 weeks (body surface area 0%). This clinical response was maintained in the following months together with controlled joints symptoms. After 192 weeks of treatment, routine colonoscopy (recommended in Italy for colon cancer screening in people over 50 years old) was performed despite the absence of any gastrointestinal symptoms. The colonoscopy showed the disappearance of the regular vascular pattern in colonic mucosa. Biopsies taken from the ascending, transversal, and descending colon revealed the presence of intraepithelial lymphocyte cells infiltrate and apoptotic bodies, the absence of distortion of the cryptic architecture and fibrosis of the basal lamina, with no signs of active and/or granulomatous inflammation. Pathognomonic signs of IBD in active phase were not observed and a diagnosis of lymphocytic MC likely of iatrogenic origin was made. Since the patient was not taking any other medication, secukinumab was considered the likely culprit for these colonic changes. Secukinumab treatment was interrupted and replaced by anti-IL-23 risankizumab, maintaining both psoriasis and PsA under control. Importantly, 12 weeks after discontinuing secukinumab, the complete resolution of histological MC features was observed. No recurrence of MC was observed after 9 months.

## Discussion

MC is a chronic inflammatory colon disease that frequently causes watery diarrhea, associated with fecal incontinence and abdominal pain. Unlike Crohn's disease and ulcerative colitis, this condition is not associated with an increased risk of colon cancer.^3^ In Europe, estimates suggest an incidence of 6.8 to 24.7 cases per 100,000 person-years.^3^ Due to its clinical features a differential diagnosis should be made with irritable bowel syndrome. In fact, MC is diagnosed in approximately 12% of patients with watery diarrhea who undergo colonoscopy.^4^ Smoking and autoimmune diseases, such as thyroiditis, polyarthritis (eg, rheumatoid arthritis) and celiac disease, are the most recognized risk factors.^2^ Non-steroidal anti-inflammatory drugs and proton-pump inhibitors are reported as the main culprits of drug-induced MC.^3^ Treatment of this form of MC form needs the discontinuation of the drug and the use of budesonide. In refractory cases, the therapy is based on tumor necrosis factor-α inhibitors, vedolizumab, or thiopurines.^3^ This case describe a MC detected following a routine colonoscopy performed for screening purposes, in a patient who underwent secukinumab for 192 weeks, but he had no bowel-related symptoms. While IL-17A is elevated in both psoriasis and IBDs in the colonic mucosal infiltrate,^4^ it is expected IL-17 inhibitors to be equally effective for psoriasis and IBDs. Conversely, a trial suggested no benefit from IL-17A inhibition with secukinumab in patients with Crohn's disease, with disease worsening in 15.4% of patients.^5^ IL-17A has a protective role towards the intestinal mucosa, as its blockade is believed to lead to an excessive inflammatory response towards the intestinal microbiota.^6^ Several cases of flare-ups or new onsets of IBDs related to the use of anti-IL-17 are reported in literature. According to a postmarketing study using Vigibase Data, 1129 gastrointestinal Individual Case Safety Reports were identified including 850 IBDs, 83.3% under secukinumab and 16.2% with ixekizumab; among the remaining 279 Individual Case Safety Reports, 79.2% undifferentiated colitis and 10.4% MC were observed (83.1% under secukinumab).^7^ However, new onset of IBD under anti-IL-17 is believed uncommon, with an incidence rate of 2.4 cases per 1000 patient-years. A systematic review with meta-analysis conducted by Yamada et al found no increased risk of new-onset IBD with the use of anti-IL-17 agents compared to placebo, although it remains unclear to what extent this is attributed to treatment or to underlying disease.^8^ Referring to our case, it is interesting that MC was diagnosed in a patient who had been treated with secukinumab for 192 weeks and who was completely asymptomatic. Existing literature described symptomatic secukinumab-induced MC cases with abdominal pain and diarrhea, as the 1 reported by Gandu et al.^9^ Further developments are needed to evaluate the eventual appropriateness of screening and follow-up programs in patients undergoing therapies with anti-IL-17. Fries et al suggested a detailed family history and fecal calprotectin dosage in selected patients undergoing anti-IL-17 treatment, estimating the cumulative incidence of new-onset IBD at approximately 1% in patients treated with secukinumab.^10^ Fecal calprotectin is typically elevated in IBD and is used to differentiate them from irritable bowel syndrome in order to reduce the number of invasive examinations such as colonoscopy.

The reported case adds an extra element to the case history of colitis induced by anti-IL-17, as the diagnosis of MC was made in an asymptomatic patient. Further research is needed to improve our knowledge about the potential link between anti-IL-17 and drug induced MC.

## Conflicts of interest

None disclosed.
